# Papass clinical trial protocol: a multi-component school-based intervention study to increase acceptance and adherence to school feeding

**DOI:** 10.1186/s12889-019-7988-2

**Published:** 2019-12-05

**Authors:** Rafael Lavourinha Pinto, Bárbara da Silva Nalin de Souza, Anna Beatriz Souza Antunes, Mara Lima De Cnop, Rosely Sichieri, Diana Barbosa Cunha

**Affiliations:** 1 Department of Epidemiology, Social Medicine Institute, State University of Rio de Janeiro, Rua São Francisco Xavier, 524, 7° andar, bloco E, sala E 7017B, Maracanã, CEP 20550-900 Rio de Janeiro, Brazil; 2 Department of Collective Health, Collective Health Institute, Federal University of Mato Grosso, Cuiabá, Mato Grosso Brazil; 3 Gastronomy course, Institute of Nutrition Josué de Castro., Federal University of Rio de Janeiro, Rio de Janeiro, Brazil

**Keywords:** Healthy eating, Early intervention, School, Students, Eating behavior

## Abstract

**Background:**

One of the largest school feeding programs in the world is the National School Feeding Program of Brazil. However, results from the 2012 National School Health Survey indicated that only 22.8% of 9th grade students in Brazilian public school system consumed school meals. The literature presents few studies aiming to promote healthy food consumption in the school environment from interventions, which found inconclusive results. Thus, this study aims to present a protocol to evaluate the effectiveness of multi-component school-level interventions to increase adherence and acceptance to school feeding.

**Methods:**

School-based multi-component clinical trial with students from 4th-9^h^ grade from 3 municipal schools of Sumidouro, Rio de Janeiro, Brazil, in 2019. The study design will be parallel, with 3 arms: Control group (without intervention); Intervention group 1 (changes in school environment) and Intervention group 2 (changes in menu and school environment). Interventions in the environment will be based on the principles of choices architecture and, the modification in the dishes that make up the menus offered to the students, on the factors that contribute to poor adherence and acceptance to school feeding, identified by focus groups. Adherence to school feeding will be assessed through a specific question in the questionnaire directed to the frequency of consuming school meals in the week, applied by researchers in three moments. Acceptance will be assessed from the acceptability test application with dishes served to students during the year. Statistical analyses will be performed using generalized linear models, which will be used to assess the impact of the intervention, and will include 3 main variables: intervention, time and the intervention x time interaction.

**Discussion:**

This study will investigate if the impact of the implementation of interventions in the environment and in the dishes served to students may increase adherence and acceptance to school feeding. Positive results could show the effect of implementing interventions throughout Sumidouro’s public school system, as well as throughout the country, aiming to improve the consumption of school meals.

**Trial registration:**

Brazilian Registry of Clinical Trials, RBR-7mf794. Date of registration: December 27, 2018.

## Background

The world has made progress in reducing extreme poverty in the last few years. In 2015, there was a reduction of over a billion people living in this situation, in comparison to 1990. Progress was driven by strong global growth and the growing wealth of many developing countries [1].

Although an economic advance on the world stage is observed, the United Nations Food and Agriculture Organization has published in its report that since 2015 the number of undernourished people has increased. In 2018, more than 820 million people worldwide were still hungry. Among these, 34,7 million were located in Latin America, highlighting the great challenge of meeting the goal number 2 of the Sustainable Development Goal (SDG), which predicts zero hunger by 2030 [2].

This scenario is more disturbing when looking at the population of students. The United Nations World Food Program estimates that, throughout the world, 66 million people go to school hungry [3]. School feeding programs are the most prevalent social protection network in the world [4]. It is a recognized strategy for improving student nutrition and health, as well as increasing access to education and school attendance, reducing inequalities in education and improving students’ performance. When linked to local agriculture, school feeding also strengthens the region’s economic development by cooperating with small producers [5].

In Brazil, the National School Feeding Program (PNAE) was created in the 1950s and is characterized as the country’s longest-running public policy on food and nutrition security, being considered one of the largest, most comprehensive and long-lasting school feeding programs in the world, only behind to India [5–7]. According to the World Food Program report, more than 40 million children and adolescents benefit daily from PNAE in Brazil [6]. In 2019, the Brazilian federal government had a budget of 3.5 billion reais (approximately 877 million dollars) to support school meals in basic education, serving students in kindergarten, elementary, high school and youth and adult education. This amount is passed on directly to the states and municipalities (executing entities), that complement it with their own resources [8].

However, providing food allowance for students enrolled in the public school system does not guarantee that adherence to the program is satisfactory in Brazil. Results from the 2012 National School Health Survey (PeNSE) indicated that only 22.8% of 9th graders in public schools consumed school meals at least 3 days a week [9]. In a new edition from PeNSE, conducted in 2015, revealed that only 31.3% of Brazilian students enrolled in the 9th grade of public schools consumed school meals on at least 3 days of the week [10], being classified as low adherence to school feeding [11].

Acceptance of school meals (approval of the dish offered) is another important factor, once it reflects the quality of services provided by schools and refers to the average preference of foods offered by them [12]. According to the National Education Development Fund (FNDE), menus must be prepared by nutritionists, based on students’ eating habits, and should have 85 to 90% acceptance [12, 13]. Silva et al. (2013), in a study conducted with 1448 students from the state school system of Minas Gerais, Silva et al. (2013) observed that the effective acceptance, answers considered as “excellent” and “very good”, reached only 28.8% [14].

In an integrative literature review, which aimed to identify the factors that influence the adherence and acceptance to school meals among Brazilian students, it was observed that, the most relevant ones were socioeconomic variables, nutritional status, age, presence of competitive foods in the school environment, behavioural factors such as changes in dietary practices and choices, as well as inadequate preparation for eating habits [15]. In this context, the school has the role of educating, as well as making individuals aware of the countless possibilities of healthy consumption, more attentive to their practices and eating habits [13, 16–18].

Systematic review indicated that school-based interventions have the potential to improve dietary behavior. Most studies provided nutrition education to promote healthy diet, but the most effective intervention programs have multiple components. Only five studies conducted with multi-component interventions showed positive effects, such as increased preferences for healthy food and decreased daily consumption of sweetened carbonated drinks, fast food eating behavior score, frequency of fast food consumption in general and in schools, besides the reduction in fried food consumption, soda intake, and snacks high in fat, sugar, and salt [19].

Previously, Van Cauwenberghe et al. had reported the moderate evidence that educational interventions conducted by the teachers in adolescents could improve dietary behavior and inconclusive evidence of effect of environmental interventions that consisted of adapting or increasing the availability of healthy food [20]. According to Verstraeten et al., few interventions targeted the school environment, which is pointed as a challenge because of the type and complexity of changes required [19]. So, due to the complex nature of eating behavior, multicomponent interventions may contribute to influence the food supply, acting as a promising population-based approach to promote health. Furthermore, improving the community food environment is important, especially in low-income areas that present increased access to unhealthier foods [21].

Choices architecture based strategies is a tool that can change behaviour and lead children’s and adolescents’ choices for a healthier life [22]. The concept of choices architecture can be defined as an intervention that preserves freedom of choice, without coercion or obligation, only influencing people’s behavior in a predictable way for decision making [22, 23]. In recent years, there has been an increase in both interest and importance in changing children’s eating behavior for healthier choices [24]. In the school environment, these strategies can be implemented in cafeterias, making it convenient to eat healthy foods [25]. However, there are few studies in the literature that aimed to promote the consumption of healthy food in the school environment through interventions based on choices architecture, and such studies found inconclusive results [26].

Thus, studies are needed to evaluate intervention strategies to increase adherence and acceptance of school feeding. Considering that adolescents are highly susceptible to environmental influences, it is suggested that interventions carried out in the school feeding environment and in the dishes served to the students present themselves as an appropriate tool to promote an increase in adherence and acceptance of school feeding.

## Methods

### Study design

This is a multi-component school-based, clinical trial called PAPASS, which means “Professionals and Students for Healthy Eating in Sumidouro”. PAPASS is a study aimed at increasing adherence and acceptance of school meals, which will be conducted in the 2019 school year in 3 public schools in the city of Sumidouro, Rio de Janeiro, Brazil. All students from 4th to 9th grade, morning and/or afternoon shifts from the 3 selected schools will be invited to participate in the study.

**Fig. 1:**
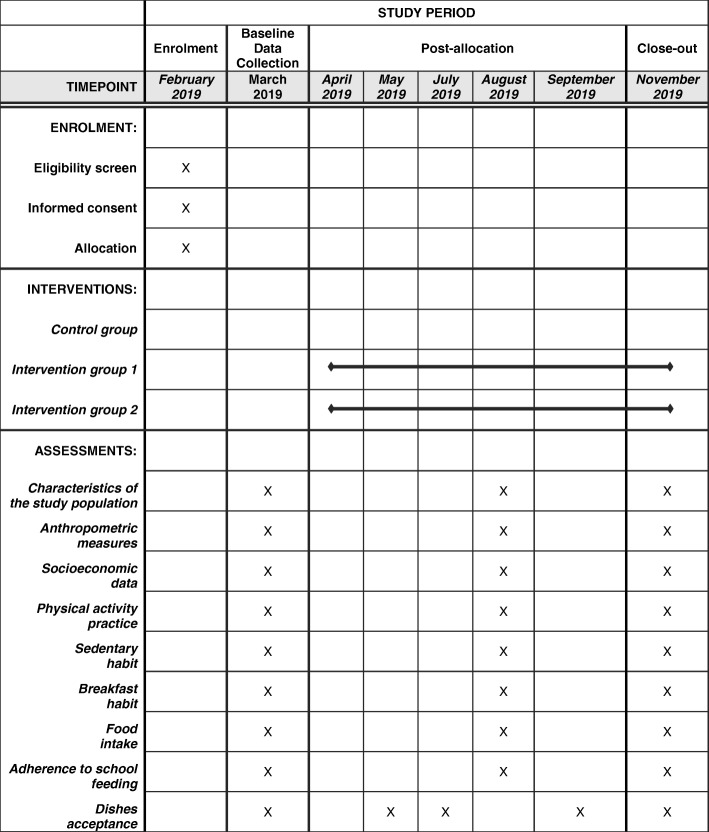
Schedule of enrolment, interventions and assessments

The study design will be parallel, with 3 arms: (1) Control group (without intervention); (2) Intervention group 1 (changes in the school environment) and (3) Intervention group 2 (changes in the menu and school environment) (Fig.1). To allocate interventions, schools will be numbered and randomly allocated to each group using opaque envelopes by people who are not involved in PAPASS.

This study was approved by the Ethics Committee of the Institute of Social Medicine (State University of Rio de Janeiro), under opinion number 2.825.207 and registration number in the CAAE Brazil Platform: 92307118.7.0000.5260 and registered in the Brazilian Registry of Clinical Trials (ReBEC) for randomized controlled trials under code RBR-7mf794 (http://www.ensaiosclinicos.gov.br). The SPIRIT recommendations were followed to describe the study protocol [27]. The complete checklist can be reviewed in Additional file 1. The signed Informed Consent Form will be obtained from all participants’ guardians and can be reviewed in Additional file 2.

### Sample size calculation

The sample size of the PAPASS study was calculated based on the results of the last edition of National School Health Survey, conducted in 2015, which revealed that only 37.21% of Brazilian students, from 6th to 9th grade, from public schools, morning or afternoon shifts, that reported the availability of school meals, consume them at least three times a week [10, 28]. Considering a 50% increase in adherence to school meals, 80% power and a significance level of 5%, a sample size of 109.4 adolescents per arm was estimated, totaling a sample of 328.2 students. Considering losses and the rate of refusal, the total sample size required was increased by 20%, raising to approximately 132 students in each arm and a total of 396 students in the sample.

### Setting and participants

This study will be conducted in the municipality of Sumidouro, which has an estimated population of 14,920 inhabitants, is located 174 km from the city of Rio de Janeiro, state capital, and is part of the Rio de Janeiro State Center Fluminense mesoregion [29]. The municipality has a Municipal Human Development Index (MHDI) around 0.611, the worst from the state, far from state capital, which has a MHDI of 0.799, ranking first in the state [30].

For this study, three of the four Sumidouro districts were included and, from the 23 municipal public schools, with classes from 4th to 9th grade, 3 of them were selected to participate in the study. All students regularly enrolled in these schools were considered eligible for this study (Fig.1). Exclusion criteria was students with physical/cognitive disabilities and pregnant adolescents.

### Data collection at school

Data on the characteristics of the study population (age, gender and skin color/ race), anthropometric measures, socioeconomic data, physical activity practice, sedentary habit, breakfast habit, food intake and adherence to school feeding will be collected in three moments (baseline - March, half of the school year – August, and end of the school year - November), using a structured questionnaire applied by trained researchers. Acceptability tests will be performed bimonthly, in order to include all menus established in the schools participating in the study.

Adherence will be assessed through a specific question in the questionnaire directed to the frequency of school meals consumptionin the week, with the options: never, once a week, twice a week, three times a week, four times a week or all days. Students who consume the food offered 3 or more times a week will be considered as adhering to the school feeding program [9]. Each adolescent will be assessed in 3 moments: at baseline - at the beginning of the school year; the second - at half of the school year; and the third - at the end of the school year. In order to avoid follow-up losses, schools will be visited 4 times at each moment of data collection (Table 1).

**Table 1:** Intervention components, description and assessment

Intervention components	Description	Assessment
Adherence to school meals	- Placement of posters on cafeteria walls and displays on tables with messages and/or images that encourage students to make healthier food choices; - The implementation of the use of tablecloths in the cafeteria with color images of fresh foods, accommodation of the fruits served in the school feeding in a prominent position, improving their visibility in an attractive way; - The equipment of the cafeterias, with the installation of a thermal distribution counter to guarantee the autonomy of food choice by the students, through a self-service system; - Placement of a banner with the daily dishes menu at the entrance of the cafeteria; application of footprints on the floor that playfully direct the adolescents to the cafeteria; - Changes in the dishes that make up the menu of the only school which will receive the two interventions (Intervention group 2).	A question about the frequency of school meal consumption, applied in 3 moments (baseline, half of the school year and end of the school year).
Acceptance of dishes offered		Application of acceptability tests applied after the 4th week of implementation of each new menu (5 in total) in 2019 and a questionnaire with questions about each food group applied in 3 moments (baseline, half of the school year and end of the school year).

To assess the level of dishes acceptance, the acceptability test will be applied for all dishes that make up the school feeding menus, as proposed by the Manual for Application of the Acceptability Test, published by the Ministry of Education [12]. The Department of School Food of Sumidouro establishes that each menu must be worked for a period of 2 months. The first set up menu (valid for February and March) will be the same as for all schools in the municipal school system. The other menus effective for the months of April–May, June–July, August–September and October–November will be differentiated among the schools that are part of the study. Therefore, the acceptability tests will be applied in March, May, July, September and November 2019. The schools allocated to the control group and intervention group 1 will continue to receive the same menus made available to the other schools in the municipality, while the school allocated to intervention group 2 will receive the new menus prepared by the research group.

The acceptability test will be applied to students who access the school cafeteria and consume the food offered. The test will be performed on cards with hedonic scale, a validated instrument which consists in the evaluation of a product - in this case, the menu - from a graded scale, with points representing the student’s acceptance of food, differentiated based on the grades (4th and 5th grade - mixed facial hedonic scale; 6th to 9th grade - verbal hedonic scale) [31, 32]. In these assessments, the student may select the expressions “I loved”, “liked”, “indifferent”, “did not like” or “hated”. When it comes to the mixed facial hedonic scale, besides these expressions, they have pictures of feelings faces. Answers will be counted for each scale expression shown on the card. If the sample has a percentage greater than or equal to 85% in the expressions “I loved it” and “I liked it”, it will be considered that the dish/food produced good acceptance. Acceptability tests will be performed in all 3 schools participating in the study, being on the 4th week of implementation of each new menu in the school of intervention group 2 and in the same period in the schools of control and intervention groups 1 (Table 1).

The rating of the socioeconomic level will be performed by applying the Brazil 2018 Economic Classification Criterion, with the questions included in the questionnaire, that refers to the amount of assets that the family owns (automobile(s), personal computers(s), dishwasher(s), refrigerator(s), freezer(s), washing machine(s), DVD player(s), microwave oven(s), motorcycle(s) and clothes dryer(s)), domestic servant(s), bathroom(s) and if have a Public Utility Services (piped water and paved street) [33].

Age will be calculated from the difference between the date of questionnaire application and the student’s date of birth.

For skin color, at the time of the interview, the student will be asked how he or she declares to be their color or race, among the following answer options: white, black / black, brown / *mulata* / brunette, yellow (oriental) / indigenous.

Weight and height will be measured using standard procedures [34]. Portable weight scales (Tanita®BC-558) will be used for weight measurements. Height will be measured using a portable stadiometer (Alturexata®), with a width of 200 cm and a variation of 0.1 cm. Two measurements will be taken and a maximum variation of 0.5 cm between them will be allowed. If the variation exceeds this value, the measurements will be repeated. The average of the two valid measures will be considered for the analysis. The classification of the nutritional status of the students will be performed according to the classification of the World Health Organization [35].

To assess the physical activity level, we used questions included in the 2012 National School Health Survey (PeNSE), which refer to the frequency and time spent in three different activities (commuting to school, physical education classes at school and extra-school physical activities), in relation to the week prior to the interview, to estimate the accumulated physical activity in the period. Adolescentss will be classified as active (≥300 min per week), insufficiently active (150–299 and 1–149 min per week) and inactive (without physical activity) [9].

Food consumption will be assessed by a short food frequency questionnaire (FFQ) (23 items), which consists of a reduced version of a validated FFQ for adolescents from Rio de Janeiro [36], with the following options: less than once a month or never; 1–3 times a month; Once a week; 2–4 times a week; 5–6 times a week; once a day; 2 or more times a day.

Sedentary habits will be assessed in two separated questions based on the number of hours on a typical weekday (Monday to Friday) spent watching television and, using a computer or playing video games, which showed the following options: < 2 h; 2–4 h; 4–6 h, 6–8 h; > 8 h. The sedentary behavior indicators adopted are based on common PeNSE 2012 questions and were based on the number of daily hours allocated to these activities [9]. The habit of having breakfast will be assessed from a question taken from PeNSE 2012, which refers to the frequency in which the student usually consumes the first meal of the day, with the following options: never or almost never; once or twice a week; 3 or 4 times a week; 5 or 6 times a week; every day [9].

In order to promote retention of study participants, telephone calls will be made with the school board requesting that students be aware of the date when data collection will happen, minimizing the number of absentees. Furthermore, meetings will be held with students’ parents and staff from each of the participating schools to raise awareness of the importance of participating and authorizing students participation in data collection. To maximize the data collected, each school will receive 3 visits, on consecutive days, to get information from students who, for some reason, may have been absent on the previous data collection.

### Intervention

The interventions will take place during the 2019 school year, beginning in April, in the two schools allocated to the intervention groups and will combine two strategies: modifications in the school environment and modifications in the menu offered by the school. Interventions in the environment will be based on the choices architecture principles proposed by Thaler & Sustein [23, 37, 38], and the interventions in the menu will be based on the factors that contribute to poor adherence and acceptance of school feeding, identified through a previously conducted focus groups with the lunch ladies (school food service employees) of the all 3 participating schools and the students of the school that will receive the two interventions (intervention group 2).

The focus groups were conducted in the second semester of 2018, lasting 2 days. On the first day, it was held with the students, and on the second day, with the lunch ladies. The focus group with the students was conducted by 1 mediator and 2 observers with number of participants ranging from 8 to 10 people [39–43]. It was guided by questions that addressed the perception about the frequency of eating school meals; the most accepted foods; the least accepted foods; the environment where the meal is consumed; the way of preparation and distribution service; as well as prior knowledge of the dishes that would be served during the week. The focus group elaborated for the lunch ladies was conducted by 1 mediator and 1 observer with number of participants ranging from 8 to 10 people [39–43]. It was contemplated with questions about: self-knowledge about the role of lunch ladies in the school; the perception of adherence and acceptance of food by the students; motivation of students for not consuming dishes; form of preparation and distribution of meals; and what actions could be taken to make school feeding more attractive to students. The activity lasted approximately 1 h and 20 min, distributed according to the complexity of each topic to be discussed. After transcribing the participants’ speeches, content analysis was performed by trained researchers in three stages, which enabled the identification of some factors responsible for not consuming school meals [44]. In face of the reports observed during the focus groups with the lunch ladies and students that allowed the identification of dishes with less acceptance among the students, it was observed that the lack of autonomy in the composition of the dish by the students generated dissatisfaction, as well as a waste of the prepared foods due to the standard portioning service performed by the lunch ladies.

Intervention group 1 will receive changes in the school environment. Modifications in the environment, based on the choices architecture, which will consist of making posters that will be fixed to the cafeteria walls, and displays, with images and messages that encourage students to make healthier food choices, that will be placed on the tables; use of tablecloths on cafeteria tables with color images of fresh foods; accommodation of the fruits served in the school meals in a prominent position, improving their visibility in an attractive way; creation of a fruit stand (common in street markets in Brazil); placement of a banner with the menu of daily dishes at the entrance of the cafeteria; application of footprints on the floor that playfully direct adolescents to the cafeteria. Changes in the meals distribution system will be made through the installation of a thermal distribution counter, which will guarantee the autonomy of the students’ choice of dishes, through a self-service system.

. Intervention group 2 will receive the same interventions as intervention group 1, with the addition of the modification in the dishes that make up the menus offered to the students, based on the reports observed during the focus groups.

The new dishes and the menus were developed by gastronomy researchers and by the nutritionist, responsible technician for the PNAE in Sumidouro, with common foodstuffs for all municipal schools in Sumidouro, acquired by bidding process. The new dishes were intended to be more palatable, favoring the addition of fruits and vegetables in the menu, as well as reducing the use of ultra-processed foods, sugar and oil. Local eating habits were respected and all requirements proposed by PNAE resolutions for the creation of school feeding menus were met [13]. It is important to point out that the new dishes suggested by the PAPASS project were previously approved by the School Food Department of Sumidouro.

A Practical Guide was developed for the lunch ladies, composed by notions of hygiene, storage and preparation techniques of the recipes that will be served in the 2019 school year. Also, sixty-one technical cards composed of quantity of ingredients, preparation method, necessary utensils, yield and portion for each dish were prepared and included in the guide. The dishes were previously tested in an experimental kitchen to correct the necessary quantities of inputs and to check the utensils that would be used, as well as the improvement of the techniques needed to prepare the new recipes. The cookery workshops with the lunch ladies were elaborated from their previous report, through qualitative research conducted from a focus group, aiming to meet the demands for new preparation techniques, especially among the less consumed foods by the students. They were conducted by trained gastronomy researchers and took place on two consecutive days in the very kitchen of the school where the lunch ladies are placed. The creation of the technical cards enabled the development of 4 lunch menus, composed by side orders, protein dish, vegetables and/or salad, dessert and drink, destined only to the intervention group 2 school.

The control group will continue to receive the standard treatment previously offered to all schools in Sumidouro’s municipal school system, without any loss to students.

## Primary and secondary outcomes

The primary outcome of the study will be adherence to school feeding, obtained from the answers given by the students in question about the frequency of school meals consumption through 1 week, applied by the researchers in three moments (baseline, half of the school year and by the end of the school year). The secondary outcome will be the acceptance of school feeding through the acceptability tests referring to the dishes inserted in the menus established in 2019 in the schools participating in the study, applied after the fourth week from the beginning of each menu. (Fig.1).

All data collected in this study will be entered in duplicate and managed using Epi-Info software, version 3.5.1 (CDC, Atlanta, GA, USA). Access to information will be limited to designated members, such as researchers. Paper documents such as consent forms, questionnaire answer sheets and anthropometric measurements will be stored on a hard disk.

## Statistical analysis

Statistical analyses for each outcome will be performed using generalized linear models that takes into account repeated measurements and missing data. The models will be used to assess the impact of the intervention and will include 3 main variables: intervention (intervention 1, intervention 2 or control school), time (treated as continuous, in months) and the intervention x time interaction. Data analysis will be performed using the SAS OnDemand for Academics (SAS Institute Inc., Cary, NC, USA).

## Discussion

Intervention strategies to promote healthy food consumption in the school environment, based on choices architecture, which include changes in the environment, such as the use of messages and images of incentives and changes in the positioning of foods offered to students [45–47] and in the presentation of foods, by offering pre-cut fruits, using containers with attractive colors and by creating playful names for dishes [46, 47], have been conducted in developed countries with inconclusive results in modifying eating behaviors [26]. In the current study, a multi-component intervention based on the theory of choices architecture and results observed from focus groups will be tested, with the aim of increasing adherence and acceptance of the food served in the municipal schools of Sumidouro-RJ, Brazil.

In Brazil, the National Education Development Fund (FNDE), responsible for the PNAE, established the application of acceptability tests by the executing entities as one of the procedures for quality control of the food served to the students [48, 49]. The following situations predispose the application of the acceptability test: whenever introducing new food in the menu, or any other innovative changes regarding the preparation, or to assess the acceptance of frequently practiced menus. Acceptance of menu dishes by the students is an important factor to determine adhesion frequency to PNAE [50] and the application of the acceptability test becomes more evident when a large variation in adherence rates is observed (from 38 to 62%) [13, 51–53].

Despite the wide range in relation to the consumption of school meals, food supply through the PNAE is associated with increased consumption of fresh foods and lower consumption of ultra-processed foods [53, 54]. In addition, regular consumption of school meals is associated with a better quality habitual diet, especially among those students with higher social vulnerability risk [55]. This can be attributed to PNAE restrictions on the use of ultra-processed foods such as processed meat, can-food, sugary drinks and concentrated and/or prepackaged foods, to the minimum fruits and vegetables supply determination three times a week and also to the use of at least 30% of the financial resources to purchase foodstuffs that come from family agriculture, which contributes not only to social development, but also to local economy [13].

To date, no Brazilian studies have been identified in the scientific literature that have been conducted aimed at changes in the school environment and in the dishes that make up the menu offered to students from the public school system, with the aim of increasing adherence and acceptance to school feeding. To this end, the controlled trial of PAPASS multi-component intervention described in this protocol document will provide data on the effects of new intervention approaches, as well as combination of interventions.

We plan to disseminate the findings from this trial through: a community presentation to the participants involved in the study, presentations at relevant conferences for researchers and healthcare professionals, as well as in peer-reviewed publications.

The hypothesis that new approaches can contribute to increase adherence and acceptance of school meals by students, if accepted, will attest to the effect of implementing interventions throughout the public school system of Sumidouro, as well as the country, with the objective of improving the food consumption of the students.

## Supplementary information

**Additional file 1.** Proposed Extended SPIRIT Checklist.

**Additional file 2.** Informed Consent Form sent to all parents authorizing the participation of students in the study.

## Data Availability

The datasets generated by the current study are not publicly available, but are available from the corresponding author on reasonable request.

## Abbreviations

FNDE: Portuguese abbreviation of Fundo Nacional de Desenvolvimento da Educação
MHDI: Municipal Human Development Index
PAPASS: Portuguese abbreviation of Professionals and Students for Healthy Eating in Sumidouro
PeNSE: Portuguese abbreviation of National School Health Survey
PNAE: Portuguese abbreviation of National School Feeding Program
ReBEC: Portuguese abbreviation of Brazilian Registry of Clinical Trials
SDG: Sustainable Development Goal
