# Supplementary material for: Papass clinical trial protocol: a multi-component school-based intervention study to increase acceptance and adherence to school feeding
Source: BMC Public Health. 2019 Dec 5;19:1644. doi: 10.1186/s12889-019-7988-2 (PMC6896593; doi:10.1186/s12889-019-7988-2)
Supplement: Supplementary file 2 — Additional file 2. Informed Consent Form sent to all parents authorizing the participation of students in the study. [file 12889_2019_7988_MOESM2_ESM.doc]

**CONSENT TERM**

You and your child are being invited to participate, as volunteers, in the research entitled “Impact of intervention aimed at greater acceptance and adherence to school feeding: a randomized community-based trial in Sumidouro - RJ”, conducted by Rafael Lavourinha Pinto. This research aims to evaluate the impact of interventions performed in the school environment aiming at greater acceptance and adherence to school feeding.

You have been selected because your child is enrolled in the school that will be part of the research. The activities will take place at the school itself. Your participation is not mandatory. At any time, you may withdraw from participation and withdraw consent. Your refusal, withdrawal or withdrawal of consent will not cause harm.

Your participation in this research will consist of: assessing your child's school feeding through questionnaires, in which you will be asked questions about your child's eating habits and questions as to whether your son or daughter usually engages in any kind of physical activity. Weight and height will also be measured at the beginning and end of the study. All these measurements will be performed by trained researchers. You will be asked to participate through a socio-economic questionnaire, where questions will be asked about the head of household's education, the items you have in your home (refrigerator, television, etc.) and whether you have running water and asphalt in your street. With this research, your son or daughter will benefit from receiving information about healthy eating, as well as the result of the anthropometric measurements performed.

As with any scientific research, participants in this research are subject to minimal risks such as discomfort when subjected to weight and height measurements. Because it is a voluntary participation, the research will not be paid, as it will have no cost for students and guardians. Any participation expenses will be paid or reimbursed by the research.

The data obtained through this research will be confidential and will not be disclosed on an individual level to ensure the confidentiality of your participation. This means that no researcher or assistant will provide any information about your or your child's data.

The responsible researcher undertook to publicize the results obtained in academic and scientific circles in a consolidated manner without any identification of participating individuals.

If you agree to participate in this research, sign the end of this document, which has two copies, one of them being yours, and the other, of the responsible researcher / research coordinator. Following are the phone numbers and the institutional address of the responsible researcher and the Research Ethics Committee - CEP, where you can answer your questions about the project and your participation, now or at any time.

Contacts of the responsible researcher: Rafael Lavourinha Pinto, nutritionist, at the Institute of Social Medicine of the Rio de Janeiro State University, Rua São Francisco Xavier, 524, room E 7017-B, e-mail: rlavourinhap@gmail.com, telephone: 2334-0235, extension 269, or (21) 97922-5465 to facilitate communication.

Research Ethics Committee of the UERJ Institute of Social Medicine: Rua São Francisco Xavier, 524 - room 7.003-D, Maracanã, Rio de Janeiro, Zip Code 20550-013, telephone (21) 2334-0235, extension 211. Email : [cep.ims.uerj@gmail.com](mailto:cep.ims.uerj@gmail.com).

I declare that I understand the objectives, risks and benefits of my participation in the research, and agree to participate.

Sumidouro, ________(date)_________.

Student’s name: ___________________________________________

Signature of the responsible: ________________________________

Researcher’s signature: __________________________________
